# A Study of Drop-Microstructured Surface Interactions during Dropwise Condensation with Quartz Crystal Microbalance

**DOI:** 10.1038/srep35132

**Published:** 2016-10-14

**Authors:** Junwei Su, Majid Charmchi, Hongwei Sun

**Affiliations:** 1 Department of Mechanical Engineering, University of Massachusetts Lowell, MA, USA

## Abstract

Dropwise condensation (DWC) on hydrophobic surfaces is attracting attention for its great potential in many industrial applications, such as steam power plants, water desalination, and de-icing of aerodynamic surfaces, to list a few. The direct dynamic characterization of liquid/solid interaction can significantly accelerate the progress toward a full understanding of the thermal and mass transport mechanisms during DWC processes. This work reports a novel Quartz Crystal Microbalance (QCM) based method that can quantitatively analyze the interaction between water droplets and micropillar surfaces during different condensation states such as filmwise, Wenzel, and partial Cassie states. A combined nanoimprinting lithography and chemical surface treatment approach was utilized to fabricate the micropillar based superhydrophobic and superhydrophilic surfaces on the QCM substrates. The normalized frequency shift of the QCM device together with the microscopic observation of the corresponding drop motion revealed the droplets growth and their coalescence processes and clearly demonstrated the differences between the three aforementioned condensation states. In addition, the transition between Cassie and Wenzel states was successfully captured by this method. The newly developed QCM system provides a valuable tool for the dynamic characterization of different condensation processes.

Water condensation on solid surfaces is a critical process for a wide range of industrial applications, such as steam power plants^1^, water desalination^2^,^3^, water harvesting^4^, thermal management^5^,^6^ and anti-fog surface^7^ development. Dropwise and filmwise condensations are the two major condensation regimes. For the filmwise condensation, a liquid film is formed on the cold solid surface. This liquid layer behaves as a significant barrier for both heat and mass transfers; and thereafter, results in a lower heat transfer coefficient and slower rates of condensation. Whereas, dropwise condensation, first reported by Schmidt *et al*.^8^, where the generated droplets have diameters in the range of a few nanometers to agglomerations visible to a naked eye, is free of the thermal and mass barrier limitations associated with filmwise condensation. As a result, dropwise condensation can potentially offer orders of magnitude higher condensation efficiency than that of filmwise condensation, and therefore, it is very attractive for industrial applications. In fact, it has been reported that DWC produces heat transfer coefficients 5 to 7 times larger than the values found in filmwise condensation under the same experimental conditions^9^.

Different superhydrophobic surfaces have been developed to achieve dropwise condensation in the last decade^10^,^11^. Several fabrication methods have been reported to produce biomimetic roughness-induced superhydrophobic surfaces, such as plasma-enhanced chemical vapor deposition^12^, conventional photolithography and etching^13^, self-assembled monolayers on nanostructures^14^, template-based extrusion^15^, electrospinning^16^, and some other techniques such as a slippery liquid-infused porous surface^17^, and layer-by-layer deposition^18^. Some of these surfaces have shown a great potential to further improve the efficiency of dropwise condensation^19^.

When a liquid drop is placed on a rough hydrophobic surface, two distinct wetting states could occur: (A) the Cassie (or Cassie-Baxter) state in which drop is suspended on top of the roughness; and (B) the Wenzel state, where the liquid penetrates downward into the roughness without spreading, as shown in Fig. 1A,B. Note that both Cassie and Wenzel states could exhibit high contact angles; however, the penetrated liquid portion of the Wenzel state produces a high contact angle hysteresis. In addition, it was observed that a metastable state–partial Cassie state could exist during the dropwise condensation depending on the thermal, physical and chemical conditions of the surface^20^,^21^, as illustrated in Fig. 1C.

Currently, the common approach adopted to study DWC is based on an indirect method, in which the heat flux and temperature of the condensation surfaces are measured and presented in the form of either heat flux or heat transfer coefficient vs. sub-cooling temperature^19^,^22^,^23^. Although it is a straightforward evaluation method for heat transferring during DWC, some detail information, such as drop growth mechanism and wetting state, could not be revealed by this practice. Therefore, some droplet dynamic analyses were performed with the assistance of direct optical observation with spatial resolutions of a few micrometers^21^,^24^,^25^. For example, Narhe and Beysens described sequential condensation stages on square shape micro-pillars by an optical microscope and a CCD camera^24^. Dorre and Rühe observed the transition from Wenzel to Cassie states during DWC with similar method^21^. Later, Environmental Scanning Electron Microscope (E-SEM) with about 10 nm spatial resolution and 10 to 50 frames per second temporal resolution was successfully used to observe the dropwise condensation process^19^,^26^,^27^,^28^. Rykaczewski illustrated the different growth mechanisms of individual water droplets on a superhydrophobic surface with an *in-situ* E-SEM technique^26^. More recently, Bhattacharya *et al*. developed *in-situ* Transmission Electron Microscopy (TEM) technique to study the nucleation dynamics of water nanodroplets during condensation with 0.1 nm resolution^29^. The small field of view and high resolution of these microscopic observations make them only suitable for viewing the nucleation or individual drop growth dynamics of the dropwise condensation on a focused surface area. In addition, if the contact angle of the droplet is larger than 90 degree, the top view captured by the microscope can not reveal the true liquid/solid interface by the projected area (see Fig. 2). Therefore, there is a great need for the development of a new tool that can provide the real-time characterization of liquid/solid interaction during dropwise condensation on a global scale.

Quartz crystal microbalance (QCM), is a simple, cost-effective, and high-resolution sensing device, that relies upon the piezoelectric effect to sense a mass loading change on its surface with an extremely high sensitivity, (i.e., less than 10 ng/cm^2^)^30^,^31^. A traditional 10 MHz QCM typically consists of a thin (≈170 μm) quartz disk where its both sides are coated with a thin layer (≈100 nm) of gold serving as its electrodes. Because of the crystal orientation of the quartz and its piezoelectric properties, when an alternating voltage is applied to its electrodes a transverse shear wave will result on the substrate surface. The resulting shear wave has a frequency influenced by a number of factors that will be discussed in later sections.

QCM devices were first used to characterize the viscoelasticity of a wide variety of polymer materials^32^,^33^. Traditionally QCMs were employed in a vacuum or in a gaseous environment, later Nomura showed that a crystal material, completely immersed in a liquid, can be driven to oscillate in a stable manner^34^. This finding extended the QCM application to include liquid phase environment^35^,^36^. The proof of QCM effectiveness in both gaseous and liquid phase environments provided an opportunity for investigating different condensation processes. Combined with the optical microscopic technique, QCM device can serve as a complementary tool to quantitatively characterize the liquid/solid interactions during condensation processes on different surfaces.

In this work, first, several condensation experiments on a flat surface were conducted to validate the QCM methodology. Polymethyl methacrylate (PMMA) micro-scale pillar structures were then fabricated on one side of the 10 MHz QCM by employing nanoimprinting lithography (NIL) technology^37^. Then, the hydrophobic or hydrophilic surface properties were achieved by different chemical treatments of the micropillars for condensation study.

The QCM output frequency signal depends on the liquid/solid contact state. Therefore, the frequency shift of the QCM output signals can be an indicator of a condensation regime. The output signal of QCM device, for different condensation states, were recorded by a frequency counter and processed in the form of normalized frequency shifts. Based on the theoretical relationships, the output signals were processed and different condensation regimes were identified. In addition, the transitions from Wenzel to Cassie or partial Cassie states were detected on superhydrophobic micropillar surface. With this novel characterization method, this study intends to answer the following critical questions related to DWC such as: the wetting state during condensation on micropillar surfaces–is it in Cassie, Wenzel, or partial Cassie state? If it is in partial Cassie state, how much of the surface area would stay in Cassie state? Is there any transition between Wenzel and Cassie states during dropwise condensation? How long the hydrophobicity of a surface can be maintained during dropwise condensation?

## Theory

### Measurement Mechanism of QCM

The resonant frequency shift of a QCM device is linearly correlated with the rigid mass deposited on the quartz surface as described by Sauerbrey’s equation^38^:


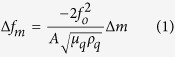


where, Δ*f*_*m*_ is the resonant frequency shift due to the deposited mass Δ*m* on the sensor, *f*_o_ is the excitation frequency of the resonator (10 MHz), *A* is the sensing area (gold surface) of the quartz crystal, called the electrode surface (0.2 cm^2^), *ρ*_*q*_ is the density of the quartz crystal (2.648 g/cm^2^), and *μ*_*q*_ is the shear modulus of the quartz crystal (2.947 × 10^11^ dyn/cm^2^).

**Figure 2:**
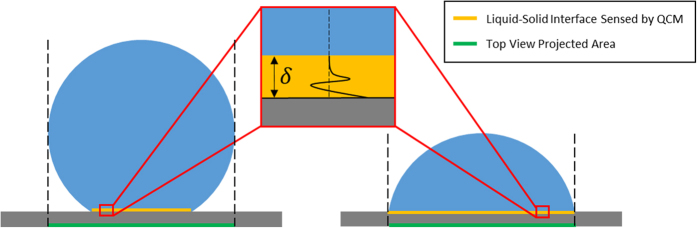
Schematic of the comparison between top view projected area observed by microscope and liquid/solid interface sensed by QCM on different surfaces. The inset is the resonant shear acoustic wave decaying within propagation thickness at liquid/solid interface.

When operating in a liquid, a thin evanescent shear wave, having a propagation thickness *δ*, will be generated near the liquid/solid interface, (see the illustration in Fig. 2). In this situation, the entire surface of the sensing area (*A*) is covered by liquid, (referred to flooded condition). Solving the wave propagation and the Navier-Stokes equations simultaneously, the resonant frequency shift (Δ*f*_*flood*_) and the shear wave propagation thickness (*δ*) can be determined by the Kanazawa equation^39^:


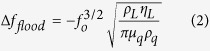



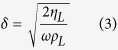


where, ω = 2*πf*_0_ is the angular frequency, *η*_*L*_ and ρ_*L*_ are the dynamic viscosity and the density of the liquid, respectively. For water as working fluid, the wave decay length, *δ*, was estimated to be 180 ± 20 nm for the 10 MHz QCM sensor used in this research.

Previous investigations revealed that when a QCM sensor was exposed to a liquid, the frequency shift of the sensor corresponded to the effective mass of the liquid layer that represented in half of the wave decay thickness (i.e., *δ*/2)^39^. Denoting liquid/solid interfacial area by *A*_*L*_, the effective mass of liquid deposited on the sensor can be obtained by:


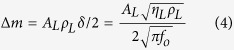


Introducing Eq. 4 into Sauerbrey Eq. 1, and knowing that the added mass is due to the effective liquid mass, it will yield to:


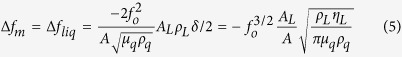


Under flooded condition, where *A*_*L*_* = A*, and *Δf*_*liq*_* = Δf*_*flood*_, Eq. 5 becomes identical to Kanazawa Eq. 2. In the general case, the frequency shift due to liquid loading could be expressed by Eq. 6.
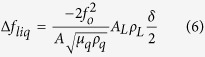
As can be seen, all parameters appear in Eq. 6 have fixed values except for *A*_*L*_. Hence, the measured frequency shift of the QCM is directly proportional to the global liquid/solid interfacial area, (shown as amber lines in the cross-sectional representation of droplets in Fig. 2).

Optical microscope imageries have been used to estimate liquid/solid interfacial area. However, careful image processing is required to obtain the correct values, if the system permits. As illustrated in Fig. 2, when the contact angle of the drop is smaller than 90° (right droplet), the projected area of the drop on the substrate observed by an optical microscope (green line) is equal to the liquid/solid interfacial area of the drop on the substrate, depicted by the amber line. Whereas, for the case of larger than 90° contact angle, the direct optical image (green line) overestimates the actual liquid/solid interfacial area. Hence, for DWC on superhydrophobic surfaces, optical imagery may not be a reliable measuring technique. The new QCM methodology investigated here provided a remedy to the optical shortcoming.

### Micro Pillar Design

Aided by the experimental observation and interfacial free energy calculation, researchers proposed design guidelines for superhydrophobic surfaces intended for dropwise condensation applications^28^,^40^,^41^. For water vapor condensing on pillars, the ratio of Cassie and Wenzel state energies, (E*), is related to the advancing contact angles of the Cassie and Wenzel states, as follows: for advancing Cassie state, the relation is 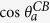 = −1; and for the advancing Wenzel state, we have 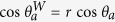; and therefore, the energy ratio E*, expressed by Eq. 7 can be used to determine the drop morphology^28^.
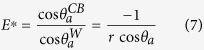
where, *r* = 1 + *πdh*/*l*^2^ is the dimensionless surface roughness, *d* and *h* are the diameter and height of the pillars, respectively, and *l* is the center to center spacing between pillars. Also, *θ* is the droplet contact angle and the subscript “a” denotes advancing, the superscript “CB” refers to Cassie-Baxter state, and the superscript “W” indicates Wenzel state.

When E^*^ > 1.0, the Wenzel state is energetically favorable. On the other hand, when 0 < E^*^ < 1.0, Cassie state would take place. The dimensions of micropillars used in this research were chosen according to this principle. In addition, the static intrinsic and apparent contact angles of Wenzel and Cassie drops were measured and presented in Table 1 26,41.

## Experimental Methodology

### QCM Surface Preparations

**Figure 3:**
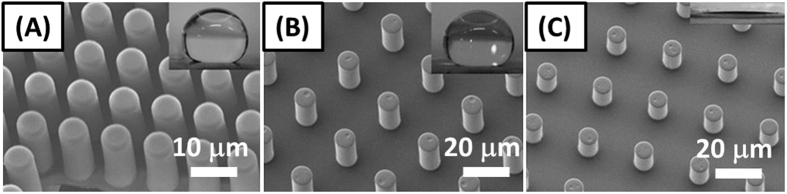
SEM images of PMMA micropillar arrays (tilted 20 degrees) for (**A**) partial Cassie condensation-S4; (**B**) Wenzel wetting condensation-S2; (**C**) filmwise condensation-S1.

A hot embossing based fabrication technique-nanoimprinting lithography (NIL), was used to fabricate polymer micropillars on standard AT-cut 10 MHz QCM substrates. The fabrication details can be found in a previous work^37^. Figure 3 provides the SEM (JEOL JSM 7401F) images of the micropillars for three condensation modes–(A) partial Cassie condensation (surface #S4), (B) Wenzel wetting condensation (S2), and (C) filmwise condensation (S1). The inset presented in each image of Fig. 3 is the corresponding static apparent contact angle droplet image for each case.

For data presentation, two baselines are considered. One is the case of flooded surface, whereas, the second case refers to a dry surface (no condensation). The flooded surface was prepared through oxygen plasma (Harrick PDC-32G) treatment of micropillars for 3 minutes followed by QCM measurement in water before the plasma treatment lost its effect in about 30 minutes. The dry baseline was obtained by directly operating QCM device in the air.

Different condensation states were achieved by treating the micropillar surfaces with the following steps:

1. For superhydrophilic surface (S1), the micropillar surface was treated with oxygen plasma and was utilized immediately without further modification;
2. For Wenzel wetting surface (S2), micropillars were coated with a molecular layer of perfluoro silane using molecular vapor deposition (MVD) in a vacuum chamber for an overnight process period;
3. For partial Cassie surface (S4), micropillars were first coated with a 5 nm gold layer using a sputtering machine (Denton Vacuum Desk IV) and then immersed in a 5 mM 1 h, 1 h, 2 h, 2 h-perfluorodecanethiol/ethanol solution for 24 hours to obtain a continuous Self-Assembled Monolayer (SAM) on pillar surfaces^42^;
4. For hydrophobic flat surface (S3), a smooth, gold-plated surface was used (measured contact angle was about 69°).

### QCM Based Experimental System

**Figure 4:**
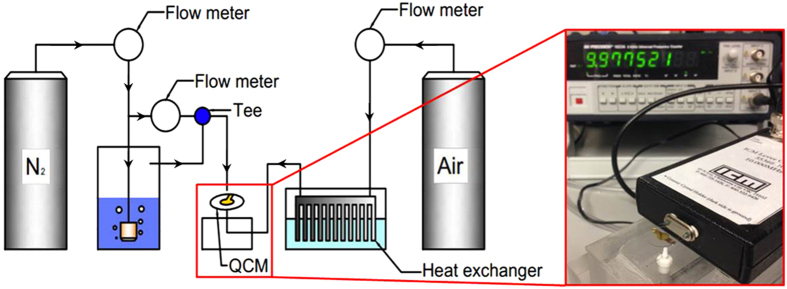
Schematic of the QCM based measurement system.

Figure 4 presents a schematic diagram of the QCM based condensation measurement system. As seen there, it mainly consisted of a vapor generator, a substrate cooling system, and a frequency shift measurement units. The QCM had a diameter of 13.7 mm, its electrode diameter was 5.11 mm, and its thickness was 0.17 mm.

For the vapor generation unit, dry nitrogen, supplied from a cylinder, was passed through a bubbler to produce vapor saturated nitrogen at room temperature. The saturated nitrogen is then combined with another stream of dry nitrogen to obtain a desired Saturation Point (SP, defined as the ratio of the vapor pressure to the saturation pressure of the vapor associated with the surface temperature^28^, ranged 1.02–1.72). Two flow meters were used to monitor and control the flow rates of dry and vapor saturated nitrogen streams.

To cool the condensation surface without inducing external vibration to QCM, a small amount of cold air, (3 L/min), supplied from a compressed air cylinder, was used. The air was first passed through a heat exchanger that was cooled by liquid nitrogen. The cold air was directed to the back of the condensation surface placed on the QCM. During the experiments, the QCM was maintained at 8 °C as monitored by two type E thermocouples.

The QCM and its accompanied condensation surface were oriented horizontally. The QCM was driven by a Lever Oscillator (ICM35366-10, Oklahoma) and the resonance frequency was monitored and recorded by a frequency counter (BK 1823A, Fotronic Corp.). The frequency data was recorded every 0.25 second (i.e., 4 Hz) and analyzed by a built-in LabView program. Simultaneously, optical images of condensation were captured with an optical camera (AmScope MU500) attached to a microscope (Olympus ML-26) or a trinocular microscope (AMScope). The optical characterization would not only show the drop motion, but also provide the assistance to better understand the QCM signal. The detailed discussion of each case will be shown in later sections.

### Signal Processing

A normalized frequency shift, Δ*F*, is defined to reflect the relationship between the measured frequency shifts of QCM, Δ*f*_*liq*_, and condensation states, as follows:


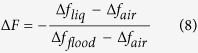


where, Δ*f*_*flood*_ represents the QCM frequency shift when the whole sensing surface of QCM is covered by water layer, (flooded baseline), and Δ*f*_*air*_ is the frequency shift when the device operates in air, (dry baseline). Hence, the dimensionless value of Δ*F* ranges from −1 to 0, for flooded and dry conditions, respectively. This normalized frequency shift data was used to quantitatively determine the state of condensation on different surfaces. The QCM results were also compared with the results obtained from optical imageries. The comparison showed that for some surfaces excellent agreement exists; whereas, for partial Cassie condensation regimes, optical imagery failed to produce the meaningful result for the liquid/solid interaction.

As stated earlier, experimental data recorded 4 times per second and therefore, some interesting higher frequency events such as droplet jumping on superhydrophobic surfaces^22^,^25^, occurring within a very short period of time (~ of ms) were not captured by the existing data acquisition system. Employing a 1 KHz or higher rate data acquisition system can significantly enhance the capacity of the experimental apparatus for analyzing such phenomena in the future. In this study, however, due to the presence of micropillars, the probability of droplet jumping was insignificant.

## Results and Discussion

### Typical Data Presentation and Method Validation

**Figure 5:**
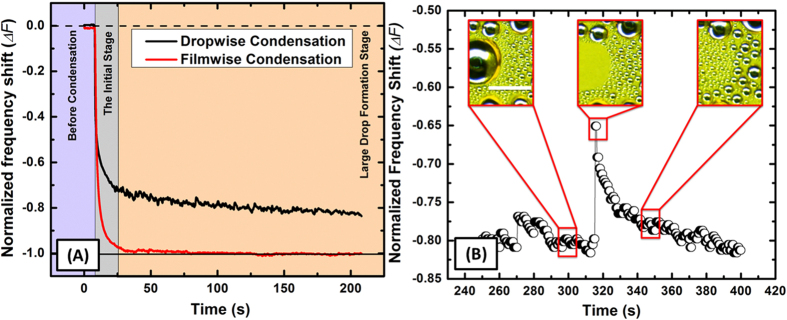
(**A**) Resonant frequency shift signal of dropwise and filmwise condensation on flat surfaces: (i) before condensation, (ii) the initial stage of condensation, and (iii) the large drop formation stage; (**B**) The detail QCM frequency signals and correlated microscopic images about interfacial area fluctuation (The scale bar of the inset images is 1 mm).

The condensation process can be divided into two distinct stages in addition to a “pre-condensation” stage. Figure 5A illustrates typical normalized frequency shifts, (Δ*F*), curves for drop- and film-wise condensations on flat hydrophobic and hydrophilic surfaces, respectively. At first, the frequency shift shows zero value corresponding to the dry baseline condition, while a dry nitrogen stream was flowing over the surface. When the stream was switched to a moist nitrogen, Δ*F* decreased sharply indicating that nucleation was taking place –microscale droplets were being generated and were growing but, without significant droplet interactions at the initial stage. As shown in Eq. 6, the interfacial contact area between the droplets and the sensor surface is the main reason for frequency shifts. After the nucleation, the droplets grew and some droplets coalesced with the neighboring ones. As time passed, the condensation process transitioned to the second stage referred to “large-drop formation stage” in which the normalized frequency shift approached to a relatively steady value. The accompanied Fig. 5B illustrates significant signal fluctuations in the second stage for dropwise condensation. The optical microscope images revealed that some large drops were removed by gas flow from the hydrophobic surface, resulting in sudden decreases of liquid/solid interfacial area and hence, raising the frequency shift (see Fig. 5B). Subsequent to the large drop removal, a new cycle of drop generation started, liquid/solid contact area increased, and as a result, the frequency shift slowly decreased.

On the other hand, when the surface was exposed to oxygen plasma for hydrophilic property behavior, filmwise condensation regime was produced and the liquid fully covered the sensor surface causing a flooded condition as shown by the red curve in Fig. 5A.

To further verify that the normalized frequency shift of QCM is directly proportional to the liquid/solid interfacial area, a series of experiments were carried out on the flat hydrophobic surface with different saturation points (*SP*) of the moist stream (defined as the ratio of the vapor pressure to the saturation pressure associated with the sensor surface temperature^28^). Note that, the presence of non-condensable gases, such as nitrogen, induced a diffusion resistance on the condensation surface and reduced the droplet growth rate and the number of nucleation sites^43^.

**Figure 6:**
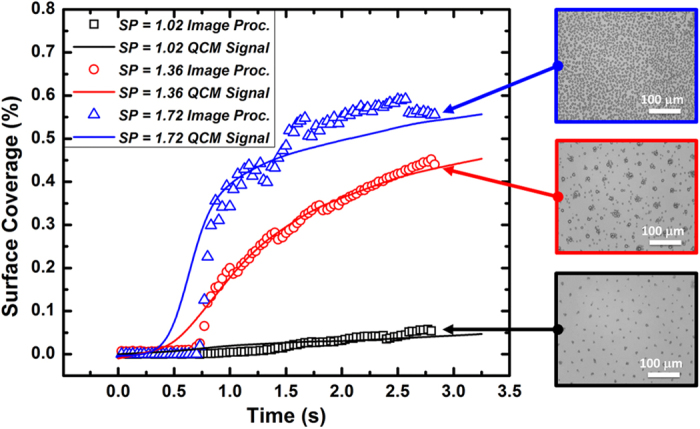
Comparison of drop-surface interface obtained from direction observation (optical microscope) and QCM measurement for different saturation points (*SP* = 1.02, 1.37, and 1.72).

In these sets of experiments, optical images of dropwise condensation at the initial stage on the flat hydrophobic surface were recorded, analyzed using ImageJ software, and compared with data obtained by QCM system. The fractions of the sensing surface covered by the uniformly distributed droplets were obtained from the image processing and from QCM frequency shifts and both outcomes are compared in Fig. 6. As shown there, results for three different saturation points are presented and in all three cases, the QCM frequency shift signals are in excellent agreement with the corresponding data acquired from images. It should be pointed out that the contact angles of drops were less than 90°, therefore, the top view images provided by the optical system rendered reliable liquid/solid contact area.

### Condensation on Microstructured Surfaces

**Figure 7:**
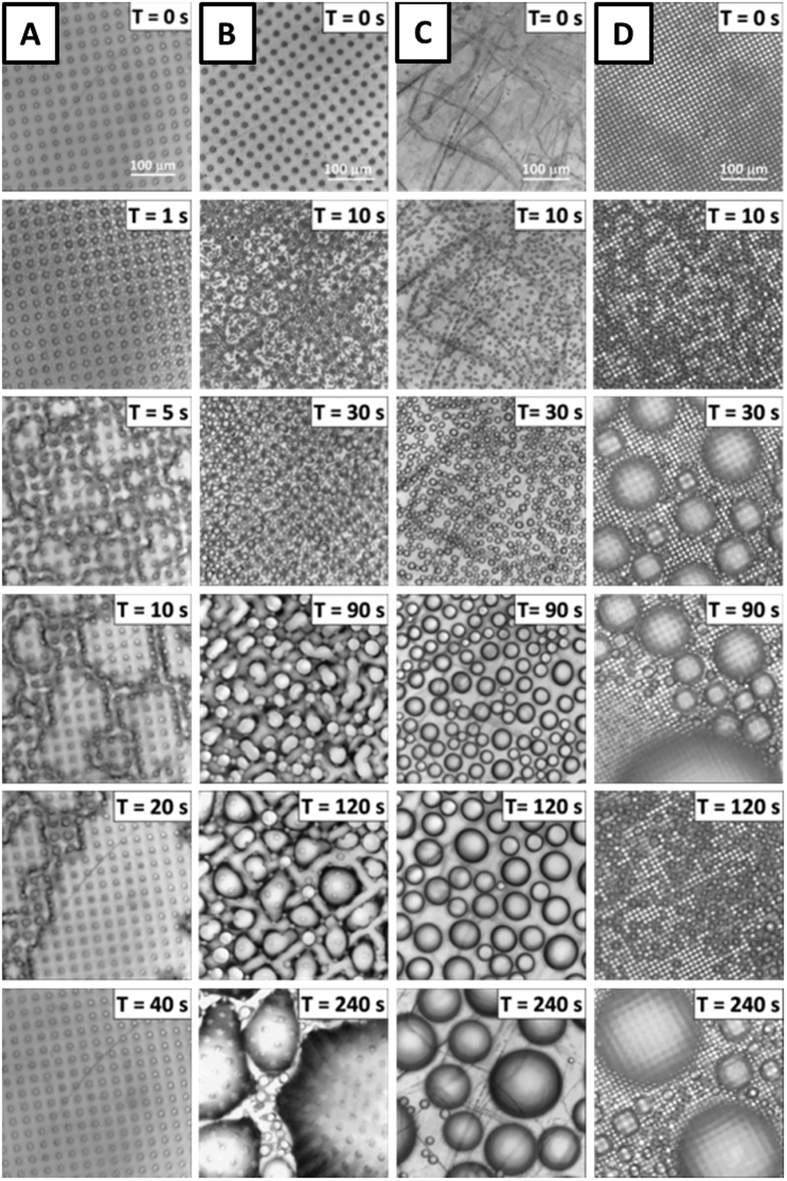
Time advancing optical microscopic images of condensations on different surfaces at SP = 1.72: (**A**) filmwise condensation on a hydrophilic micropillar surface (S1), (**B**) Wenzel dropwise condensation on a hydrophobic micropillar surface (S2), (**C**) dropwise condensation on a flat hydrophobic surface (S3), and (**D**) partial Cassie dropwise condensation on a hydrophobic micropillar surface (S4).

**Figure 8:**
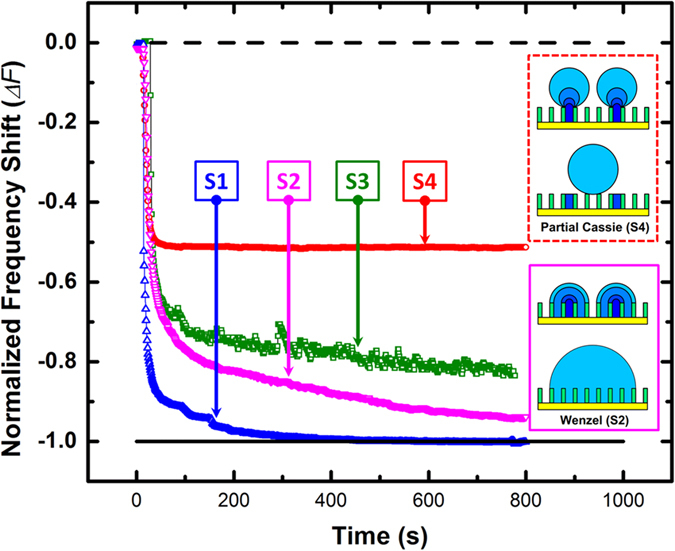
Normalized frequency shift signals of condensations on different surfaces (*SP* = 1.72). The insets are the droplet growth mechanisms of Wenzel and partial Cassie DWC.

Four different condensation scenarios were investigated with the QCM technology for the saturation point of 1.72. They were: (A) filmwise condensation on a micropillar surface (hydrophilic surface S1, see Fig. 3C); (B) Wenzel dropwise condensation on a micropillar surface (S2, Fig. 3B); (C) dropwise condensation on a flat hydrophobic surface (S3); and D) partial Cassie dropwise condensation on a micropillar surface (S4, Fig. 3A). Figure 7 presents top-view of the time-lapse microscopic images of the four condensation regimes on different surfaces and the corresponding normalized frequency shifts are displayed in Fig. 8.

#### Filmwise condensation on a superhydrophilic micropillar surface (S1)

Column A of Fig. 7 presents the optical microscopic images for condensation on the superhydrophilic micropillar surface S1. In the initial period, time = 0 to 1 s, the nucleation of droplets took place on the entire surface, (i.e., on the top and the sidewalls of the pillars, as well as on the base area in between the micropillars). In the next time period, (time = 1 to 5 s), the droplets grew between pillars and the drops merged into irregular liquid films. For time >5 s, the liquid films started to merge until the entire surface was flooded. For this case, the QCM response is shown in Fig. 8 by the blue curve. As seen there, the normalized frequency shift starts at zero (dry condition) and if one follows the progress of the QCM frequency shift for surface S1, it clearly reveals that the QCM signals quickly reached the flooded baseline as soon as the continuous water film covered the whole sensing surface.

#### Dropwise condensation on Wenzel micropillar surface (S2)

Column B in Fig. 7 shows the images of Wenzel dropwise condensation on the micropillar surface. Unlike the case for filmwise condensation, most of the droplets were nucleated between pillars within 30 seconds of a time period, followed by the coalescence of the droplets into larger drops pinned in the spaces between pillars as can be seen in images for time = 90 to 120 s. As time passed, the meniscuses of the drops became curved and then irregular as the droplets coalescence into larger drops, (shown in time = 240 s image), until the entire surface was almost fully covered by a few large Wenzel drops–approaching flooded condition.

The pink line in Fig. 8 presents the QCM frequency response for the Wenzel dropwise condensation. After the initial stage of nucleation and droplet growth, the frequency shift gradually approaches to near complete wetting condition, unlike the sharp decrease in frequency shift seen in the case of filmwise condensation (surface S1). Close analyses of QCM response combined with the microscopic images provide a confirmation on a reasonable correlation between the normalized frequency shift signals and the droplets were pinned between micropillars during the growth^44^ resulting in the strong contact angle hysteresis. In the large drop formation stage, the drops only keep growing and coalescing without drop removal. As a result, the spaces in between the micropillars were gradually filled with water, which was responsible for the gradual decrease in normalized frequency shift. By comparing the microscopic images in Fig. 7 and the initial stages in Fig. 8, this experiment also confirmed that the initial nucleation rate on a hydrophilic surface is usually higher than the rate on a hydrophobic surface. A lower free energy barrier requirement for nucleation on hydrophilic surfaces believed to be the reason^45^.

#### Dropwise Condensation on flat surface (S3)

Column C of Fig. 7 and the green curve in Fig. 8 provide the microscopic images and the QCM signals for a typical dropwise condensation on a flat hydrophobic surface, respectively. In the initial stage, time = 0 to 10 s, similar to previous cases, nucleation and rapid droplet growth are evident and normalized frequency shift sharply drops. After about 100 seconds, the decay in frequency shift markedly slows. However, the signal is no longer smooth–fluctuating about a gradual decaying average. Since there is no pillar structure exist, the contact angle hysteresis would be much weaker than the Wenzel pillar surface. Therefor coalesced water droplets recede from originally occupied surface areas. This results in the sudden change of surface coverage and reduction of frequency shift. After these receding events, new droplets nucleate on the re-exposed surfaces, which decrease the normalized frequency shift again. In addition, it was observed that some large drops were removed from the surface by drag forces induced by flowing moist gas stream as described earlier for hydrophobic flat surfaces (see Fig. 5B). In summary, the resonant frequency fluctuation responded to the competition between droplets removing, receding and re-nucleation.

#### Dropwise Condensation on partial Cassie surface (S4)

The images of partial Cassie condensation and the QCM response are depicted in column D of Fig. 7 and red curve on Fig. 8, respectively. It can be seen that almost all the droplets in this condensation regime have the spherical shape and can potentially be removed by the gas flow, in contrast with what we observed for Wenzel dropwise condensation. For example, comparison of the drops generated on surface S2 and S4, (i.e., columns B and D), reveals two different behaviors. On surface S2, small droplets were first formed, grew and finally the drops merged with their close neighboring drops forming larger drops covering substantial portion of the phase change surface; whereas, on the surface S4, as Cassie state is the energetically favorable state, water droplets tends to grow upward, (not in transverse direction), as the nucleation sites were formed in between micropillars, shown as the insets in Fig. 8. It is important to mention that when the droplets merge, some extra surface energy will be released and as a result, the newly formed larger drop tends to lift off and obtains upward mobility on the S4 surface. As a result, the newly merged drops have a tendency to move up reducing interfacial surface contact or be removed by the gas flow leaving a few discrete wetted spots in between micropillars for subsequent generation of droplets. Time = 90 s image shows a few discrete wetted spots in between pillar spaces after the larger drops were removed. In the interval between T = 90 s to 240 s, a new generation of droplets reappearing, growing, coalescing and experiencing removal again^26^.

It is apparent that the partial Cassie dropwise condensation is a more efficient mode of condensation compared to the Wenzel dropwise regime. The frequency shift, shown in Fig. 8, quickly reached a stable value at near half the value seen for flooded baseline case. This is an indication that the hydrophobicity was maintained during the entire condensation process, partial Cassie state was in play, and the portion of the wetted surface stayed reasonably constant at about 52% of the micropillar cavities^46^. Although the top view projected area observed by optical microscope fluctuates a lot, the actual wetted spots in between micropillars remains the same, as discussed in the previous section.

### Wetting State Transition

**Figure 9:**
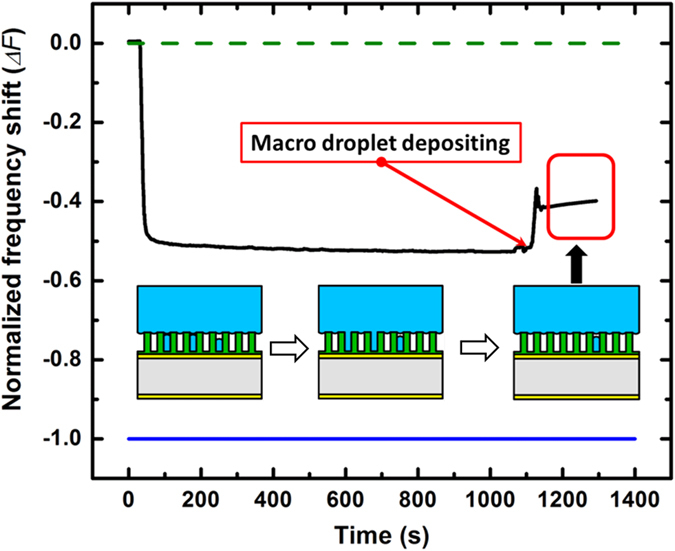
Wetting state transitions from Wenzel to Cassie on partial Cassie hydrophobic micropillar surface after DWC.

An important phenomenon in dropwise condensation on rough hydrophobic surfaces is the transition between Wenzel and Cassie states^47^. To study this phenomenon, a macro-scale drop (about 8 mm in diameter) was manually deposited on a partial Cassie pillar surface soon after a condensation experimental run. Since the Cassie state is energetically favored regime compared to Wenzel state, some of the elongated liquid existed in the wetted spots beneath the deposited drop transitioned from Wenzel to Cassie state immediately when they came in contact with the large Cassie drop. This resulted in an increment of normalized frequency shift of the QCM as presented in Fig. 9; about 13% of wetted spots transited from Wenzel state to Cassie state based on the QCM frequency shift data. For partial Cassie state surfaces, a transition from Wenzel-to-Cassie is highly possible and has been visually observed by other researchers^21^.

## Conclusion

This paper presents a QCM based technique to quantitatively measure the liquid/solid interface changes during dropwise condensation on flat and micro-structured surfaces. The micro-structured surfaces were fabricated on the bare QCM substrates by nanoimprinting lithography technique. First, series of condensation experiments were conducted on a hydrophobic flat surface to validate the methodology. For the filmwise condensation on a superhydrophilic micropillar surface, the frequency shifts of QCM quickly dropped down to the complete flooded baseline. The dropwise condensation on a hydrophobic flat surface and two micropillar surfaces were studied with a combined optical imaging and QCM dynamic signal analysis. In the condensation on a flat hydrophobic surface, the normalized frequency shift of QCM oscillated due to the changes in wetted surfaces caused by drop removal and re-nucleation. For the Wenzel condensation surface, the QCM resonant frequency gradually decreased to the flooded level due to the loss of hydrophobicity as the result of droplet coalescence and entrapment of liquid within the structure. The partial Cassie condensation showed a stable QCM frequency shift signal corresponding to about 52% of the phase change surface and discrete wetted spots remain reasonably unchanged during the continuous dropwise condensation process. The transition from Wenzel to Cassie state was quantitatively probed by manually depositing a macro-scale drop on a superhydrophobic micro-pillar surface, immediately after partial Cassie condensation. This probing effort illustrated the capacity of QCM based system for analyzing dynamics of wettability and condensation process.

## Additional Information

**How to cite this article**: Su, J. *et al*. A Study of Drop-Microstructured Surface Interactions during Dropwise Condensation with Quartz Crystal Microbalance. *Sci. Rep.* **6**, 35132; doi: 10.1038/srep35132 (2016).

## Figures and Tables

**Figure 1:**
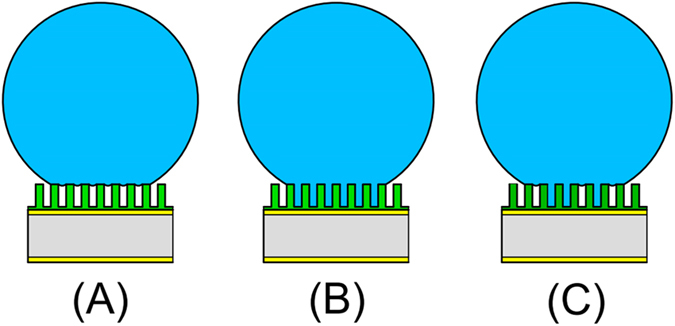
Schematic of a liquid drop under (**A**) Cassie state, (**B**) Wenzel state, and (**C**) partial Cassie state.

**Table 1:** Dimension, contact angle, and dimensionless energy for different micropillars.

	Dimensions				Contact angle (°)		E*
	*d* (μm)	*l* (μm)	*h* (μm)	*r*	Intrinsic Advancing	Apparent Static	
S1 (pillars)	10	25	15	1.75	42	5	N/A
S2 (pillars)	10	25	15	1.75	96	152	5.45
S3 (bare)	N/A	N/A	N/A	1	72	69	N/A
S4 (pillars)	7	10	22	5.84	105	149	0.66
